# An Analysis of Clinical, Surgical, Pathological and Molecular Characteristics of Endometrial Cancer According to Mismatch Repair Status. A Multidisciplinary Approach

**DOI:** 10.3390/ijms21197188

**Published:** 2020-09-29

**Authors:** Giulia Dondi, Sara Coluccelli, Antonio De Leo, Simona Ferrari, Elisa Gruppioni, Alessandro Bovicelli, Lea Godino, Camelia Alexandra Coadă, Alessio Giuseppe Morganti, Antonio Giordano, Donatella Santini, Claudio Ceccarelli, Daniela Turchetti, Pierandrea De Iaco, Anna Myriam Perrone

**Affiliations:** 1 Gynecologic Oncology Unit, Azienda Ospedaliero-Universitaria Policlinico di Sant’Orsola, 40138 Bologna, Italy; giulia.dondi@aosp.bo.it (G.D.); sara.coluccelli2@unibo.it (S.C.); alessandro.bovicelli@unibo.it (A.B.); pierandrea.deiaco@unibo.it (P.D.I.); 2 Centro di Studio e Ricerca delle Neoplasie Ginecologiche (CSR), University of Bologna, 40138 Bologna, Italy; antonio.deleo@unibo.it (A.D.L.); alessio.morganti2@unibo.it (A.G.M.); donatella.santini@aosp.bo.it (D.S.); claudio.ceccarelli@unibo.it (C.C.); daniela.turchetti@unibo.it (D.T.); 3 Center for Applied Biomedical Research, Alma Mater Studiorum-University of Bologna, 40138 Bologna, Italy; camelia.coada@unibo.it; 4 Molecular Diagnostic Unit, Azienda USL Bologna, Department of Experimental, Diagnostic and Specialty Medicine, University of Bologna, 40138 Bologna, Italy; 5 Unit of Medical Genetics, Azienda Ospedaliero-Universitaria Policlinico di Sant’Orsola, University of Bologna, 40138 Bologna, Italy; simo.ferrari@unibo.it (S.F.); lea.godino@aosp.bo.it (L.G.); 6 Laboratory of Oncologic and Transplantation Molecular Pathology, Department of Experimental, Diagnostic and Specialty Medicine, University of Bologna, 40138 Bologna, Italy; elisa.gruppioni@aosp.bo.it; 7 Radiation Oncology Center, Azienda Ospedaliero-Universitaria Policlinico di Sant’Orsola, Department of Experimental, Diagnostic and Specialty Medicine, University of Bologna, 40138 Bologna, Italy; 8 Sbarro Institute for Cancer Research and Molecular Medicine, Center for Biotechnology, College of Science and Technology, Temple University, Philadelphia, PA 19122, USA; giordano@temple.edu; 9 Department of Medical Biotechnologies, University of Siena, 53100 Siena, Italy; 10 Pathology Unit Azienda Ospedaliero-Universitaria Policlinico di Sant’Orsola, 40138 Bologna, Italy

**Keywords:** endometrial cancer, Lynch syndrome, mismatch repair genes, Lynch-like cancers, microsatellite instability, Lynch syndrome associated tumors

## Abstract

Since 2016, our hospital has applied tumor testing with immunohistochemistry (IHC) in endometrial cancer in order to detect mutations of mismatch repair genes (MMR). All cases with MMR deficiency proteins expression are sent for genetic testing, except those with *MLH1* protein deficiency, in which case genetic testing is performed if negative for promoter hypermethylation. The primary aim of this study was to investigate the ability of our algorithm to identify Lynch syndrome (LS). The Secondary aims were to investigate the relationship between MMR status and clinicopathological features and prognosis of primary endometrial cancer (EC). From January 2016 to December 2018, 239 patients with EC were retrospectively analyzed and subdivided according to MMR status. Patients were divided in three groups: MMR proficient, LS and Lynch-like cancer (LLC). LS was characterized by a lower age and BMI, more use of contraceptive and less use of hormonal replacement therapy, nulliparity and a trend versus a better prognosis. LLC appeared more related to MMR proficient than LS and exhibited a more aggressive behavior. Our multidisciplinary approach permitted a correct diagnosis of germline mutation in patients with newly diagnosis EC and it confirmed clinicopathologic and prognostic characteristics of LS.

## 1. Introduction

Endometrial cancer (EC) is the most common gynecological cancer in countries with high standard of living. Well recognized risk factors are represented by the triad of metabolic syndrome: obesity, diabetes and hypertension to which are added hormonal factors such as parity, polycystic ovarian syndrome and all causes of hyperestrogenism not balanced by progesterone/progestin presence [1]. Historically, ECs were divided in two groups: estrogens and non-estrogen related tumors. In recent years, to improve EC outcomes, traditional hormonal dualism was abandoned and a more heterogeneous disease was recognized based on genetic and molecular classification, as reported in The Cancer Genome Atlas (TCGA) and Proactive Molecular Risk Classifier for Endometrial Cancer (ProMisE) [2,3,4]. As a part of this evaluation, DNA mismatch repair (MMR) status was recommended to be investigated in all EC specimens. A compromised MMR system leads to an accumulation of somatic genetic mutations eventually resulting in carcinogenesis and involving microsatellite repeats, a condition closely related to microsatellite instability (MSI) [5]. Up to 25% of ECs demonstrate disruption of the MMR pathway, revealed by high MSI and/or loss of MMR protein expression (MMR-deficiency) by immunohistochemistry (IHC). Approximately, 13%–25% of MMR-deficient ECs are related to pathogenic germline variants in the *MLH1*, *MSH2*, *MSH6* or *PMS2* (Lynch syndrome) genes, while 62%–73% result from somatic hypermethylation of the promoter region of the *MLH1* gene [6].

Colorectal cancer (CRC) and EC are the most common LS-associated tumors (LATs) developing at earlier ages [7], followed by tumors arising to gastrointestinal, urinary and hepatobiliary tracts, ovary, pancreas, brain, sebaceous glands and skin [8]. EC functions as the sentinel cancer for more than 50% of affected women, who may present this tumor as the first manifestation at a median age of 44 years [9], preceding the diagnosis of the next tumor by 8–10 years [10]. Given the strong association between EC and LS, the screening of EC specimens for abnormalities in the MMR genes has become routine for many institutions [11,12]. On the basis of the screening approach prescribed for patients with newly diagnosed CRC, several recent international guidelines, including the 2014 guidelines from the American College of Obstetricians and Gynecologists (ACOG), the Society of Gynecologic Oncology (SGO) and the 2016 National Comprehensive Cancer Network (NCCN) guidelines recognize the value of the universal tissue testing for all patients with newly diagnosed EC [13,14,15]. Reasons in favor of expanding the screening approach are supported by many recent findings about failure of clinical criteria in identifying MMR-deficient cases for EC [16]. Almost 30% of LS patients did not meet the standards of the Bethesda Guidelines revised in 2004 [17,18,19,20] or Amsterdam II criteria [21,22]. Currently, there are numerous proposed screening algorithms since guidelines for universal screening are not yet fully established [23,24]. MMR-IHC is one of the two screening approaches (the other one is molecular MSI analysis) analyzing the expression of *MLH1*, *MSH2*, *MSH6* and *PMS2* proteins [25]. Its advantages are the ease of performance, efficiency, moderate cost and its wide availability, reasons why most modern pathology laboratories and pathologists have the expertise to properly make the analysis and interpret the results. For all patients that exhibit IHC loss of *MLH1*, a PCR-based *MLH1* promoter methylation assay is performed to discriminate the somatically hypermethylated cases from potential LS ones [26]. The confirmation phase of LS diagnosis involves a more expensive genetic testing, in the context of a genetic counseling process. The determination of the MMR status is important not only for the diagnosis of LS but also for finding prognostic factors useful to select treatments. The few studies present in the literature have given conflicting results also because they are very heterogeneous. MMR deficient status was correlated with better prognosis and response to adjuvant therapies in some studies and with poor outcomes in others. Moreover, the differences in survival and risk of recurrence between somatic and germline MMR deficiency in *MLH1*, *MSH2*, *MSH6* or *PMS2* are poorly investigated. A definition of the role of MMR status in EC is mandatory.

In this study we report a multidisciplinary experience of different Units (Gynecologic Oncology, Pathology, Molecular Diagnostics, Medical Genetics) of the University Hospital of Bologna, Italy, on the universal screening for LS in newly diagnosed EC. An algorithm inclusive of MMR IHC screening, *MLH1* hypermethylation somatic testing and germline MMR gene testing was applied for the detection of LS and Lynch-like cancer (LLC) cases.

The main aim of the study was to evaluate the correct assessment of LS in patients with EC as primary diagnosis by testing our multidisciplinary algorithm. The secondary aim was to identify clinical, surgical, pathological and oncological prognostic factors among LS, LLC and MMR proficient patients (MMR-proficient).

## 2. Results

### 2.1. IHC Analysis and MMR Protein Expression Assessment

**Figure 1:**
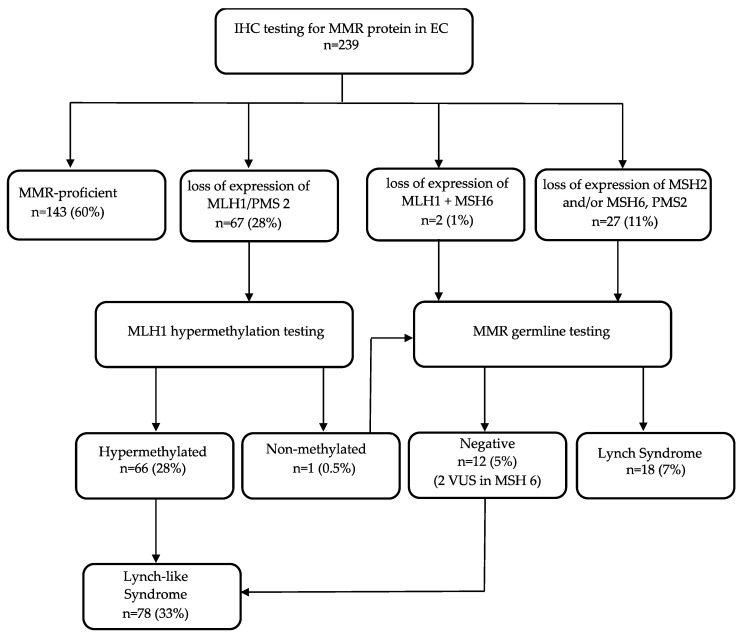
Flow chart of diagnosis of Lynch syndrome, Lynch-like syndrome and mismatch repair proficient cases in our population of endometrial cancers.

IHC-screening to assess MMR proteins expression was performed on tumor samples from 239 cases enrolled in study, after the application of inclusion and exclusion criteria. The schema is shown in Figure 1.

The analysis was carried out from 237 surgical specimen and two biopsies. At IHC analysis, 143 ECs (60%) had normal expression of MMR proteins and were classified as MMR-proficient. In the remaining 96 cases (40%) there was a lack of expression of one or more MMR proteins. Of these cases, 67 patients presented a lack of expression of *MLH1* and *PMS2*, which was associated to *MLH1* promoter methylation in all cases except one. In two cases, the loss of expression of *MSH2* was concomitant with *MLH1*. In the remaining 27 cases, there was a loss of *MSH6* protein expression associated in 24 cases with *PMS2* protein deficiency and in 13 cases with *MSH2* protein deficiency. Based on IHC analysis and promoter methylation test, 30 out of 96 patients were referred for genetic counselling and testing.

### 2.2. Germline MMR Status Assessment and LS Diagnosis

Thirty patients underwent genetic counselling and germline testing; of those, 18 were found to carry germline pathogenic variants of MMR genes and were classified as LS (Figure 1). Their clinical, pathological and genetic details are reported in Table 1.

In the remaining 12 cases, no pathogenic variants were found in the MMR genes; however, two biologically related patients had the same variant of unknown significance (VUS) of the *MSH6* gene. Excluding MMR-proficient and LS, we found 78 cases of LLC.

### 2.3. Correlation Between Clinical and Pathological Features and Germline Variants in Patients with LS

**Figure 2:**
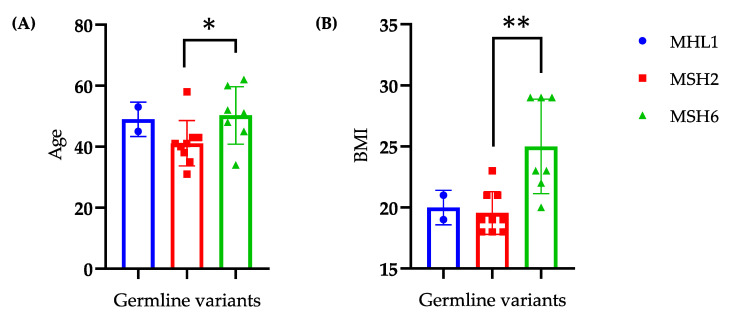
Correlation between the classes of germline variants (*MLH1*, *MSH2*, *MSH6*) and clinical features of Lynch Syndrome patients: age (**A**), BMI (**B**). Data are presented as mean ± SD. Statistical significance is specified with asterisks. (*p* < 0.05 *, *p* < 0,01 ** as determined by an unpaired Student’s *t*-test). *MLH1*: MutL homolog 1; *MSH2*: MutS homolog 2; *MSH6*: MutS homolog 6; BMI: body mass index.

A correlation analysis performed between the germline variants (*MLH1*, *MSH2* and *MSH6* genes) and the clinical-pathological characteristics found that: patients carrying *MSH6* germline variants were older at EC diagnosis and had a higher BMI when compared to *MSH2* variant carriers (*p* = 0.05, Figure 2A and *p* = 0.002, Figure 2B, respectively).

We found no correlation between each of the germline variants and FIGO stage or tumor grade. No analyses were conducted for the group with MHL1 mutations due to the small number of patients (*n* = 2).

### 2.4. Comparison Among the Three Groups (LS, LLC, MMR-Proficient) for

#### 2.4.1. Personal and Familial Cancer History 

Data reported in Table 2 show a higher frequency of family history for cancer and colorectal adenomas in LS compared to the other two groups. Personal and family history was also used to assess whether the revised Bethesda criteria were met in LS patients and could correctly diagnose LS in all cases.

The results show that, by using these criteria, the diagnosis would be missed in 4/18 patients (22%). The three groups did not differ significantly from each other regarding the personal history of cancer (*p* value = 0.068).

#### 2.4.2. Clinical Features

Analyzing age and BMI, we observed that women with LS were younger and thinner, in comparison to the other groups, while those in the LLC group were older than the others but did not differ by BMI from MMR-proficient (Table 2). The age of menopause was reported similar in the three groups, LLC used more frequently hormone replacement therapy (*p* = 0.038) while LS oral contraceptives (*p* < 0.001).

The statistical analysis on the “*Parity*” variable revealed a significant difference among the three groups but without any of them standing out due to the strict Bonferroni correction. Assuming a larger LS group, it could reach significance and show an association with the nulliparity when comparing to LLC and MMR-proficient patients.

**Figure 3:**
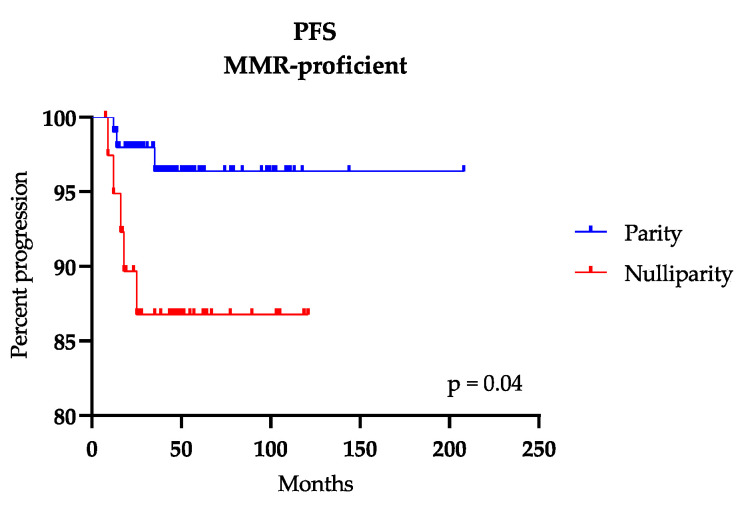
Kaplan-Meier curve for Progression Free Survival (PFS) of mismatch repair-proficient (MMR-proficient) patients according to the parity.

In order to verify whether the parity or the use of estroprogestinic therapy during life could have impacted on the recurrence rates of the three groups, we plotted a Kaplan-Meier curve, which supports the hypothesis that parity is a protective factor for MMR-proficient patients (Figure 3); likewise, LS women who had children did not have any relapse and the LLC group showed a later trend of recurrence in case of parity, although in both cases without reaching statistical significance (*p* = 0.2).

#### 2.4.3. Surgical Treatments

Surgical information is shown in Table 3.

Briefly, minimally invasive surgery was the preferred approach (69%); all patients received hysterectomy, except for two who received conservative treatment (fertility sparing), the ovaries were removed in 94% of cases, lymph node dissection and omentectomy were required in 67% and 18% of cases, respectively. Surgical data did not differ in the three groups in terms of approach and procedures, except for lymphadenectomy; MMR-proficient received retroperitoneal staging less frequently (*p* = 0.02).

#### 2.4.4. Pathological Analysis

The pathological analysis is presented in Table 3. Endometrioid was the most frequent histology in all analyzed groups. Grade 2 tumors were predominant in patients with LS and LLC, 65% and 67% respectively, while grade 1 was present in 60% of MMR-proficient. The groups of patients showed a different level of primary tumor diffusion that, in the case of a more numerous LS group, would be multifocal compared to LLC and MMR-proficient (*p* = 0.01). Increasing the number of patients could possibly yield an association between a lower LVSI with the MMR-proficient group (*p* = 0.05). FIGO stage IA was mostly represented in all population study (61%), albeit, LLC tumors tend to have more advanced stages respect the other two groups (*p* = 0.04). No differences were found between the three groups and other analyzed pathological parameters (Table 3).

In order to evaluate the concordance of histology type and grading between biopsies performed before surgery for diagnosis (Bx), frozen section during surgery (FS) and final histological analysis (His) we performed Cohen’s Kappa analysis (Table 4).

Regarding histology concordance analysis: concordance Bx-His and FS-His in the whole population study, we found a moderate agreement between the examination techniques (*k* = 0.4). For the same parameters, analyzing the three groups separately, we found that LLC reported a higher agreement between FS-His respect to Bx-His (*k* = 0.6 vs. *k* = 0.3). These parameters could not be calculated for LS and in the FS-His MMR-proficient subgroup due to the small number of patients. Regarding tumor grade concordance analysis: the concordance between Bx-His and FS-His on the whole study population was moderate-high (*k* = 0.5 and *k* = 0.7, respectively). From the analysis of the three groups separately, we found stronger agreement for MMR-proficient between Bx-His (*k* = 0.6). While the FS-His concordance is high in the whole population (*k* = 0.7) and the results for the same parameters in the LS and MMR-proficient groups are almost superimposable (*k* = 1 and *k* = 0.8).

#### 2.4.5. Postoperative Treatment and Outcome

We also considered the data of adjuvant therapies and relapses in order to define further differences between the three groups (Table 3). Women with LLC received adjuvant therapies more frequently than the other two groups (*p* = 0.01). Radiation therapy was the most used treatment. Relapse was observed in 7% of patients, mostly in the pelvic area (65%). No difference was recorded in timing and site of recurrence.

**Figure 4:**
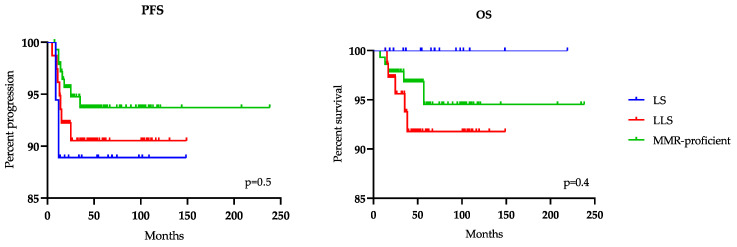
Kaplan-Meier curves for Progression Free Survival (PFS) and Overall Survival (OS) of Lynch Syndrome (LS), Lynch-like Syndrome (LLS) and MMR-proficient groups.

Our selected population of EC patients submitted to surgery had a follow-up of 53 ± 38 months (mean ± SD); Five-year OS was 96%, 10 patients died after 25.9 ± 15 months (mean ± SD) and 5-year PFS 93%, 17 patients relapsed after 15 ± 7.3 months (mean ± SD). We observed a trend for a reduced PFS and a better OS for LS patients, compared to LLC and MMR-proficient patients, although without reaching statistical significance (*p* = 0.5, *p* = 0.4 respectively) (Figure 4).

#### 2.4.6. Effects of MMR Status on Clinical and Pathological Variables

Finally, we analyzed the effect of MMR status on clinical, surgical and pathological variables and divided our population in two groups: MMR-deficient (LS and LLC) and MMR-proficient. The groups differ by BSO performed, positivity to lymph nodes, LVSI and COC intake (Table 5).

Specifically, MMR-proficient performed more frequently BSO (*p* = 0.05), lymphadenectomy (*p* = 0.01) even if without LVSI (*p* = 0.01) and received less adjuvant therapies (*p* = 0.005). On the contrary, MMR-deficient patients used more frequently COC compared to MMR-proficient (*p* = 0.038). MMR status did not impact on OS (*p* = 0.50) and PFS (*p* = 0.25, data not shown).

## 3. Discussion

Our study demonstrated that implementation with a universal screening strategy with IHC tumor testing on EC promotes the correct diagnosis of the MMR status in order to recognize patients with suspected LS. Furthermore, the division based on the MMR status in addition to the identification of the subjects at risk of developing tumors can offer a prognostic assessment that could be evaluated from the first tumor biopsy. To our knowledge, this is the first study of its kind providing a comprehensive evaluation of EC patients based on MMR status.

Our study confirms the superiority of IHC tumor testing respect to clinical criteria in screening patients with EC for LS. Using the schema in Figure 1, we correctly detected 18 patients with LS. As shown in Table 1, if we had only used clinical criteria for LS screening, we would have lost the diagnosis between 22% and 67% of cases. This becomes particularly evident in the mutation of the *MSH6* gene where 3/5 cases (60%) did not meet any of the clinical criteria. Our results confirm the current contraindications of scientific societies to use clinical criteria alone in the first diagnosis of EC due to their low sensitivity. In fact, ACOG and SGO advised that all women affected by EC should be tested with IHC and *MLH1* promoter hypermethylation before genetic testing [15]. This type of screening provides rapid diagnosis, reliability and cost reduction compared to offering the genetic test to all new diagnoses of EC.

In this series, the LS represent 7% of all EC cases; these data are consistent with the literature which reports a percentage between 2–10% [28,29]. If we consider the samples with MMR deficiency (40% of the EC) the percentage of LS rises to 18%. The MMR deficiency represents a heterogeneous population composed of three different situations: LS, hypermethylated and MMR deficient without genetic mutation (the last two groups represent the LLC patients). Therefore, these groups must be investigated as single entities, compared with each other and finally evaluated their possible prognostic implications.

In our study, we found that groups (LS, LLC and MMR-proficient) did not differ for personal history of cancer (probably because EC is a sentinel event in the population), surgical approach, histology (the majority are endometrioid), grade, lymph node metastasis, synchronous EC and ovarian cancer, type and site of recurrence; however some peculiarities must be underlined. The LS group exhibits a close anamnestic association with family history of tumors, colorectal adenomas and the Bethesda criteria and they are characterized by a lower age and BMI at the time of diagnosis of EC. Furthermore, *MSH6* germline variant showed a correlation with older age (50 vs. 41 years) and higher BMI (25 vs. 20) compared to *MSH2* (Figure 2), whereas neither stage nor tumor grade depended on the type of germline mutation as reported in literature [30,31]. As far as hormonal variables, the LS used more COCs and less HRT and were more frequently nulliparous. The first two data are difficult to be interpreted, it could be related to the younger age of LS. Regarding nulliparity, it may be due to the difficulty of conceiving of young women for hyperplastic/neoplastic alterations of the endometrial mucosa more frequently observed in patients with LS [32]. Literature data are lacking, even if nulliparity is a well-recognized risk factor associated with EC [33].

Although LS and MMR-proficient could represent the two ends of a straight line, it was interesting to observe where the LLC were placed. In CRC studies, there is no consensus whether LLC should be considered hereditary or sporadic condition, due to the risk of CRC in these patients and in their first-degree relatives is positioned between LS syndrome and sporadic CRC. Typical management of patients with LLC would not include risk-reducing surgery and screening for other LATs. In this case, cancer risk assessment would be based on personal and family cancer history [20,34]. Regarding EC, the data are even more insufficient and there is no agreement as to whether methylated cases should be included in the same class as MMR deficient non-LS. In our experience, the LLC group appeared more similar in clinical and anamnestic characteristics to MMR-proficient, in fact, no association with characteristic for LS were observed: family history of cancer, Bethesda criteria, age, BMI and parity. The pathological evaluation showed that LLC forms exhibit a more aggressive tumor as evidenced by the higher stage (*p* = 0.04), a trend towards higher grade forms and a need for more adjuvant therapies (*p* = 0.01) respect to the others two groups [35,36,37]. As a consequence, although our survival data did not reach statistical significance (probably due to the small number of patients), it may suggest a more favorable prognosis of LS patients, despite the fact that LS women seemed to develop an earlier recurrence. In general, overall survival of patients with LS-related EC did not seem to be different compared with that of sporadic EC. On the contrary, we provide interesting insight on the potentially higher aggressiveness of LLC. This data may be intriguing when compared to what has been reported by Cohen R. et al. 2017, concerning the clinical characterization of Lynch-like metastatic CRCs (mCRCs), associated with younger age, more frequent surgery for metastasis but more favorable prognosis than sporadic mCRCs [38]. This could imply a different meaning of MMR status in EC and CRC, thus more studies are needed.

Surgical data reported no difference among the three groups in terms of approach and procedures. Indeed, data in literature suggested to preserve ovaries in patients younger than 45 years, the choice to add BSO also for young patients was justified by the purpose to prevent the risk of ovarian cancer increased in patients with LS [39,40]. Although in our study patients with LS did not have a higher incidence of endometrial-ovarian synchronous tumors (11%, Table 1), in other studies, synchronous ovarian cancer was identified in about 21% of cases (in no-LS ECs, the prevalence of synchronous ovarian cancer may be approximately 4% to 5%) [41,42]. Due to this, the risk and benefits of these choices must be extensively discussed with the patient [43,44].

In order to evaluate if the prognostic factors can be taken from the diagnostic pre-operative biopsies and are maintained in the three groups, we performed a survey on the level of concordance between bx-definitive diagnosis and frozen section-definitive diagnosis for histotype and grade. The analysis showed that in all groups pre-operative biopsy had a good correspondence with final histology and grade and FS was superimposable to final histology for both parameters (Table 4). This correspondence is conserved also in LS patients even if literature reports evidences about the heterogeneity of EC and this may lead to a greater difficulty in histopathological diagnosis, especially in pre-operative biopsies [45,46]. Our data implies the possibility to correctly assess pre-operative/intra-operative risk also in LS group.

The strengths of our study include the high agreement between IHC analysis and germline testing outcomes (100%), clinical and personal data reliability, limited time gap between definitive histology and genetic testing and long follow-up offered to all study populations. Limitations of the study are the retrospective study design and low number of LS patients, which deterred us in defining a clear statistical significative difference among our groups according to some variables.

## 4. Materials and Methods

### 4.1. Study Design

This is a retrospective multidisciplinary study performed in EC patients treated at the University Hospital of Bologna, Italy, between January 2016 and December 2018. The study involved four Units: Gynecologic Oncology, Pathology, Medical Genetics and Molecular Diagnostics.

Inclusion criteria were: EC patients submitted to demolitive or conservative surgical treatment in our hospital; follow up data available at least for two years (patients that relapsed or died within two years were included); endometrial cancer specimen suitable for immunohistochemical and molecular analyses. Exclusion criteria were: specimens unsuitable for IHC and molecular analysis, lack of information on stage, histology, adjuvant treatments and genetic assessment.

Based on pathological, molecular and genetic analysis in tumor tissue, patients were subdivided in three groups: LS, patients with germline mutation of MMR genes; LLC, patients with MMR protein deficiency at IHC testing without pathogenic mutation at germline testing or patients with *MLH1* promoter methylation; and MMR-proficient, patients with normal expression of MMR proteins.

The study is a part of a larger trial on molecular classification of EC and was approved by the local Ethical Committee (CE N. 27/2019/Sper/AOUBo, approved date: 21 February 2019) and patients signed an informed consent form.

### 4.2. Data Collection

Patient data included in the study were collected through clinical records and reported in an electronic database. Clinical data reported age, BMI, hormone exposure, parity, personal and family cancer history, surgical approach and staging, adjuvant treatments and follow up data. Pathological data reported histotypes, grading, lymph-vascular space invasion (LVSI), final stage according to FIGO 2019 classification, IHC results and subsequent somatic MMR status assessment. Molecular analysis reported data about *MLH1* promoter methylation and genetics analysis reported the germline MMR status.

### 4.3. Surgical Protocol

Patients were submitted to minimally invasive surgery (MIS) or laparotomic surgery based on the surgeon’s choice, according to the standard of care [47]. Surgical staging was performed according to ESGO-ESMO guidelines [48] and included hysterectomy and bilateral salpingo-ovariectomy (BSO), with lymphadenectomy in high-grade cases and myometrial invasion greater than 50% at intraoperative frozen section (FS). Peritoneal staging was performed in serous and clear cell carcinomas. All suspected lesions were removed and analyzed. Oophorectomy could be omitted in patients younger than 45 years and myometrial invasion less than 50% confirmed at FS. Fertility sparing treatment was offered to all patients under 40 without detection of myometrial invasion by MRI and low-grade tumor. In case of up-staging in the final pathology, the patients received either a second surgical staging or radiation therapy based on the decisions of the disciplinary team on each single case.

### 4.4. IHC-Screening

All ECs were stained through IHC-screening to assess MMR proteins expression (*MLH1*, *MSH2*, *MSH6*, *PMS2*). A representative paraffin inclusion of the tumoral tissue was selected for each case and 4 sections (4 μm) where cut from the paraffin-embedded tissue and collected on pre-charged glass slides (TOMO, Matsunami Glass Ind. Ltd., Osaka, Japan). The sections were air-dried and then processed in an automated Benchmark Ultra immunostainer (Ventana Medical Systems, Tuscon, AZ, USA). The immunoreaction was visualized using the OptiView DAB Detection Kit (Ventana Medical Systems), according to the manufacturer’s protocols. The positive neoplastic population rate was evaluated through the software IMAGE Pro Plus V5.0.1, Media Cybernetics Inc., Silver Spring, MD, USA. The percentage of immunopositive cells (labelling index area; LIa) was calculated on a minimum of 30 fields at 200x for each case.

The case is considered as MMR-deficient if the percentage of positive neoplastic population is less than 1% for at least one marker, in the presence of a concurrent stromal/lymphocytic immunopositive background. Cases with inconclusive MMR (loss of the stromal/lymphocytic positive component or less than 50% of positive neoplastic cells) were subjected to microsatellite instability analysis. All other cases were MMR-proficient.

### 4.5. MLH1 Promoter Methylation

Hypermethylation testing was performed for cases with *MLH1* loss to evaluate if the deficit was acquired (hypermethylation present) or likely constitutional (absent). Approximately 100 to 200 ng of total (neoplastic and normal) DNA was subjected to bisulfite conversion using Epitect Bisulfite Kit (Qiagen GmbH, Hilden, Germany). A total of 10–20 ng bisulfite-treated DNA was carried on for PCR using the PyroMark Q24 CpG *MLH1* PCR kit (Qiagen) on a PyroMark Q24 System (Qiagen). Data were analyzed and quantified with the PyroMark Q24 Software 2.0.7 (Qiagen).

The Pyromark CpG *MLH1* kit detected the level of methylation on 5 CpG sites located in the promoter of *MLH1* gene (chromosome 3). The percentage of methylation (% of C’s present) was reported for each CpG site. A sample with methylation > 10% (mean percentage of all 5 CpG islands) was interpreted as positive, while a sample below 10% methylation was interpreted as negative.

### 4.6. MSI Molecular Testing

MSI testing was performed only in cases of abnormal or dubious MMR IHC result. Genomic DNA was extracted from fixed, paraffin-embedded (FFPE) tumor tissue (tumor-rich areas > 50%) and normal tissue using the QIAamp DNA Micro Kit (Qiagen GmbH, Hilden, Germany) according to the manufacturer’s instructions. Concentration of the extracted DNA was assessed by real time polymerase chain reaction (PCR) using the Quantifiler^®^ Human DNA Quantification kit (Thermo Fisher Scientific, Inc., Waltham, MA, USA). Microsatellite instability (MSI) analysis was performed through PCR reaction, using the CC-MSI kit (AB Analitica, Padova, Italy) according to the manufacturer’s protocol. This kit is designed to co-amplify 10 markers (BAT25, BAT26, D2S123, D5S346, D17S250, NR21, NR24, BAT40, TGFbRII and D18S58) in two separate reactions. The fluorescent amplified PCR products (from normal and tumor tissue) were analyzed by capillary gel electrophoresis on an ABI 3730XL DNA Analyzer (Thermo Fisher Scientific), using the GeneMapper software, version 4.0 (Thermo Fisher Scientific). Tumors were classified as MSI-high (MSI-H; 4/10 markers showing MSI), MSI-low (MSI-L; 1–3/10 markers showing MSI) or MS stable (MSS; no markers showing instability).

### 4.7. Genetic Counseling and Germline Testing

Women displaying *MSH2* and/or *MSH6* and or *PMS2* loss at IHC analysis of tumor tissue, as well as women with *MLH1* loss not associated to hypermethylation, were referred to Genetic Counseling.

After a comprehensive pre-test counseling session, women who gave informed consent to LS testing underwent blood sample collection. Genomic DNA purified from peripheral leucocytes isolated from a blood sample (6 mL in EDTA tube) was analyzed for mutations in *MLH1*, *MSH2* and *MSH6* genes using a homemade Next-Generation Sequencing (NGS) panel through Ion Torrent Personal Genome Machine (PGM) (Thermo Fisher Scientific). For each gene, coding exons and its contiguous intronic sequences between −21bp and +21bp were considered. Data were analyzed by the Ion Reporter Software (Thermo Fisher Scientific). Any confirmatory analysis of unclassified pathogenic mutations or variants was performed by Sanger Sequencing (Applied Biosystems, Foster City, CA, USA). Furthermore, multiplex ligation-dependent probe amplification (MLPA) analysis was performed to identify large deletions and duplications in one or more genes loss from IHC test, starting from extracted genomic DNA and using SALSA P003-*MLH1*/*MSH2* and SALSA P072-*MSH6* kits (MRC-Holland, Amsterdam, The Netherlands).

### 4.8. Statistical Analysis

Statistical analysis was performed using SPSS for Windows, version 20 (SPSS Inc., Chicago, IL, USA). Quantitative data were expressed as mean ± SD (Standard Deviation), while qualitative data were expressed as frequency and percentage. Comparison between groups was realized using Student *t*-test, Mann-Whitney, ANOVA and Chi-square tests, were appropriate. A *p*-value of <0.05 was considered significant. For multiple comparisons, the Bonferroni correction was applied. Agreement between biopsies taken from the same patient at different times: at the time of diagnosis (Bx), FS and the definitive histological medical report (His), were analyzed using Cohen’s kappa coefficient. Missing data were presented as NA (not available) in the results tables without being included in the statistical analysis. Overall, survival and progression free survival was estimated using the Kaplan-Meier analysis.

## 5. Conclusions

In conclusion, our study represents a comprehensive analysis of EC respect to their MMR status. We were able to confirm common clinical and pathological characteristics of LS; MMR-proficient represents a heterogeneous group that could be further investigated in light of new classifications (TCGA and ProMisE). Interestingly, LLC patients appear to be a distinctive group and seems to have different characteristics respect to the same group in CRC. Our study suggests that a multidisciplinary approach is necessary to assess prognostic factors in order to personalize treatments.

## Figures and Tables

**Table 1:** Clinical-pathologic and genetic characteristics of Lynch Syndrome (LS) patients.

N	Age (Years)	BMI (Kg/m^2^)	FIGO Stage	Histology	Grade	Revised Bethesda Criteria	Amsterdam Criteria	MMR IHC Pattern	Germline Variant (Pathogenicity Class) [27]
1	34	22	IIIC2	Dedifferentiated	3	Yes	No	Loss of *MSH6*	*MSH6*: c.3155_3156delAG; p.Glu1052ValfsX13 (C5)
2	41	21	IIIC1	Endometrioid	2	Yes	Yes	Loss of *MSH2*/*MSH6*	*MSH2*: c.2581C > T; p.Gln861Ter (C5)
3	40	18	II	Endometrioid	2	Yes	Yes	Loss of *MSH2*/*MSH6*	*MSH2*: deletion exon 1 to 7 (C5)
4	43	23	IA	Endometrioid	2	Yes	Yes	Loss of *MSH2*/*MSH6*	*MSH2*: deletion exon 2 (C5)
5	43	21	IA	Endometrioid	2	Yes	No	Loss of *MSH2*/*MSH6*	*MSH2*: c.484G > A; p.Gly162Arg (C5)
6	51	20	IA	Endometrioid	1	No	No	Loss of *MSH6*	*MSH6*: c.393_394delAC; p.Gln132ValfsX2 (C5)
7	45	19	IA	Endometrioid	1	Yes	No	Loss of *MLH1*	*MLH1*: c.1667 + 2T > G (C4)
8	45	29	IA	Endometrioid	2	Yes	No	Loss of *MSH6*	*MSH6*: c.1700-1701insA; p.Phe569ValfsX8 (C5)
9	35	18	II	Endometrioid	1	Yes	Yes	Loss of *MSH2*/*MSH6*	*MSH2*: c.942 + 3° > T (C5)
10 *	38	19	IA	Endometrioid	2	Yes	No	Loss of *MSH2*/*MSH6*	*MSH2*: deletion exon 15 (C5)
11 *	41	18	IA	Endometrioid	1	Yes	No	Loss of *MSH2*/*MSH6*	*MSH2*: deletion exon 15 (C5)
12	48	23	IA	Endometrioid	2	Yes	No	Loss of *MSH6*	*MSH6*: c.1869_1870insC; p.Gly624ArgfsX5 (C5)
13	53	21	IA	Endometrioid	1	Yes	Yes	Loss of *MLH1*/*PMS2*	*MLH1*: c.1409 + 2T > G (C4)
14	31	19	IA	Endometrioid	2	Yes	Yes	Loss of *MSH2*/*MSH6*	*MSH2*: deletion exon 3 to 6 (C5)
15	62	29	IB	Endometrioid	2	Yes	No	Loss of *MSH6*	*MSH6*: c.2194C > T p.Arg732Ter (C5)
16	60	23	IA	Endometrioid	2	No	No	Loss of *MSH6*	*MSH6*: c.1610_1613delAGTA; p.ys537Ilefs * (C5)
17	58	19	IA	Endometrioid	2	No	No	Loss of *MSH6*	*MSH6*: c.571_573delCTC; p.Leu191del (C5)
18	52	29	IA	Endometrioid	2	No	No	Loss of *MSH6*	*MSH6*: c.1700_1701insA; p.Phe569Valfs (C5)

* sisters; N: number; BMI: body mass index; MMR: mismatch repair; IHC: immunohistochemistry; *MLH1*: MutL homolog 1; *MSH2*: MutS homolog 2; *MSH6*: MutS homolog 6; C: Class; FIGO: International Federation of Gynecology and Obstetrics; C5: pathogenic; C4: likely pathogenic.

**Table 2:** Personal and familial oncological history of cancer and clinical data in the three groups study.

Characteristics	All Cases	LS	LLC	MMR-Proficient	*p* Value
	*n* = 239	*n* = 18	*n* = 78	*n* = 129	
**Bethesda Criteria**					<0.001
Yes	72 (30%)	14 (78%)	9 (12%)	49 (34%)	
No	155 (65%)	4 (22%)	64 (82%)	87 (61%)	
NA	12 (5%)	0	5 (6%)	7 (5%)	
**Family Cancer History**					0.002
Yes	126 (53%)	16 (89%)	32 (41%)	78 (55%)	
No	96 (40%)	2 (11%)	41 (53%)	53 (37%)	
NA	17 (7%)	0	5 (6%)	12 (8%)	
**Personal Cancer History**					0.068
Yes	38 (16%)	2 (11%)	7 (9%)	29 (20%)	
No	199 (83%)	16 (89%)	71 (91%)	112 (79%)	
NA	2 (1%)	0	0	2 (1%)	
**Colorectal Adenomas**					<0.001
Yes	12 (5%)	5 (28%)	1 (1%)	6 (4%)	
No	227 (95%)	13 (72%)	77 (99%)	137 (96%)	
Mean Age *, Years (±SD)	59.2 (±11.3)	45.5 (±9.0)	63.2 (±9.6)	58.8 (±11.1)	<0.001
**BMI Kg/m^2^, Mean(±SD)**	27.9 (±8.4)	21.8 (±3.3)	28.3 (±6.3)	28.4 (±9.5)	0.006
Mean Age **, Years (±SD)	51.3 (±4.2)	48 (±4.0)	51 (±3.5)	51.4 (±4.6)	0.212
**HRT**					0.038
Yes	32 (13%)	0	16 (20%)	16 (11%)	
No	203 (85%)	18 (100%)	62 (80%)	123 (86%)	
NA	4 (2%)	0	0	4 (3%)	
**COC**					<0.001
Yes	36 (15%)	9 (50%)	11 (14%)	16 (11%)	
No	199 (83%)	9 (50%)	67 (86%)	123 (86%)	
NA	4 (2%)	0	0	4 (3%)	
**Parity**					0.021
Nulliparous	68 (28%)	10 (56%)	18 (23%)	40 (28%)	
Parous	168 (70%)	8 (44%)	60 (77%)	100 (70%)	
NA	3 (2%)	0	0	3 (2%)	

LS: Lynch Syndrome; LLC: Lynch-Like Cancer; MMR-proficient: mismatch repair-proficient; NA: not available; SD: standard deviation; HRT: hormonal replacement therapy; COC: combined oral contraceptive; BMI: body mass index; * age at diagnosis; ** age at menopause.

**Table 3:** Surgical, pathological, adjuvant therapies and follow up data in the three groups study.

Characteristics	All Cases	LS	LLC	MMR-Proficient	*p* Value
All	*n* = 239	*n* = 18	*n* = 78	*n* = 129	
**Surgical Approach**					0.38
Minimally-invasive	165 (69%)	14 (78%)	50 (64%)	101 (71%)	
Laparotomy	72 (30%)	4 (22%)	36 (36%)	40 (28%)	
Fertility sparing	2 (1%)	0	0	2 (1%)	
**Hysterectomy**					0.51
Yes	237 (99%)	18 (100%)	78 (100%)	141 (99%)	
No	2 (1%)	0	0	2 (1%)	
**BSO**					0.07
Yes	224 (94%)	17 (94%)	77 (99%)	130 (91%)	
No	15 (6%)	1 (6%)	1 (1%)	13 (9%)	
**Staging Lymphadenectomy**					0.02
Yes	160 (67%)	14 (78%)	60 (77%)	86 (60%)	
No	79 (33%)	4 (22%)	18 (23%)	57 (40%)	
**Omentectomy**					0.75
Yes	42 (18%)	4 (22%)	12 (15%)	26 (18%)	
No	197 (82%)	14 (78%)	66 (85%)	117 (82%)	
**Histology**					0.312
Endometrioid	205 (86%)	17 (94%)	66 (85%)	122 (86%)	
Dedifferentiated	20 (8%)	1 (6%)	10 (13%)	9 (6%)	
Serous	12 (5%)	0	2 (2%)	10 (7%)	
Clear Cell	2 (1%)	0	0	2 (1%)	
**Pattern of Growth**					0.012
Focal	141 (59%)	6 (33%)	54 (69%)	81 (57%)	
Multifocal	95 (39%)	12 (67%)	24 (31%)	59 (41%)	
NA	3 (2%)	0	0	3 (2%)	
**FIGO Stage**					0.042
IA	146 (61%)	13 (72%)	37 (47%)	96 (67%)	
IB	44 (18%)	1 (6%)	22 (29%)	21 (15%)	
II	13 (6%)	2 (11%)	4 (5%)	7 (5%)	
III/IV	36 (15%)	2 (11%)	15 (19%)	19 (13%)	
**Grade**					0.331
Low	196 (82%)	17 (94%)	62 (79%)	117 (82%)	
High	43 (18%)	1 (6%)	16 (21%)	26 (18%)	
**LVSI**					0.050
Yes	71 (30%)	7 (39%)	30 (38%)	34 (24%)	
No	168 (70%)	11 (61%)	48 (62%)	109 (76%)	
**LN Metastasis**					0.220
Yes	28 (17%)	1 (7%)	14 (23%)	13 (15%)	
No	134 (83%)	14 (93%)	46 (77%)	74 (85%)	
**Tubal Lesions**					0.191
Yes	48 (20%)	1 (6%)	19 (24%)	28 (20%)	
No	191 (80%)	17 (94%)	59 (76%)	115 (80%)	
**Synchronous EC-OC**					0.360
Yes	17 (7%)	2 (11%)	3 (4%)	12 (8%)	
No	222 (93%)	16 (89%)	75 (96%)	131 (92%)	
**Endometriosis**					0.37
Yes	48 (20%)	5 (28%)	12 (15%)	31 (22%)	
No	191 (80%)	13 (72%)	66 (85%)	112 (78%)	
**Adjuvant Therapies**					0.01
Yes	113 (47%)	9 (50%)	47 (60%)	57 (40%)	
No	126 (53%)	9 (50%)	31 (40%)	86 (60%)	
**Type of Therapy**					0.17
RT	48 (20%)	5 (28%)	20 (26%)	23 (16%)	
CHT	23 (10%)	2 (11%)	5 (6%)	16 (11%)	
RT + CHT	42 (18%)	2 (11%)	22 (28%)	18 (13%)	
**Recurrence**					0.51
Yes	17 (7%)	2 (11%)	7 (9%)	8 (6%)	
No	222 (93%)	16 (89%)	71 (91%)	135 (94%)	
**Site of Recurrence**					0.53
Local (pelvic)	11 (65%)	2 (100%)	4 (57%)	5 (63%)	
Distant	6 (35%)	0	3 (43%)	3 (37%)	

LS: Lynch Syndrome; LLC: Lynch-Like Cancer; MMR-proficient: mismatch repair-proficient; BSO: bilateral salpingo-ovariectomy; FIGO: International Federation of Gynecology and Obstetrics; LVSI: lympho-vascular space invasion, LN: lymph node metastasis, EC-OC: endometrial cancer-ovarian cancer; RT: radiotherapy; CHT: chemotherapy; NA: not available.

**Table 4:** Concordance between pre-operative biopsy and final histology for histotype and grade and concordance between frozen section and final histology for grade.

Characteristics	Total	LS	LLC	MMR-Proficient
	*n* = 239	*n* = 18	*n* = 78	*n* = 143
**Histotype Concordance** **(Bx/His)**				
Yes	169 (71%)	13 (72%)	50 (64%)	106 (74%)
No	27 (11%)	1 (6%)	11 (14%)	15 (10%)
NA	43 (18%)	4 (22%)	17 (22%)	22 (15%)
*k* Cohen	*k* = 0.4	/	*k* = 0.3	*k* = 0.5
*p* value	*p* < 0.001	/	*p* < 0.001	*p* < 0.001
**Histotype Concordance** **(FS/His)**				
Yes	88 (37%)	4 (22%)	19 (24%)	65 (45%)
No	5 (2%)	1 (6%)	2 (3%)	2 (1%)
NA	146 (61%)	13 (72%)	57 (73%)	76 (53%)
*k* Cohen	*k* = 0.4	/	*k* = 0.6	/
*p* value	*p* < 0.001	/	*p* < 0.001	/
**Grade Concordance** **(Bx/His)**				
Yes	122 (51%)	9 (50%)	34 (44%)	79 (55%)
No	43 (18%)	4 (22%)	15 (19%)	24 (17%)
NA	74 (31%)	5 (28%)	29 (37%)	40 (28%)
*k* Cohen	*k* = 0.5	*k* = 0.5	*k* = 0.5	*k* = 0.6
*p* value	*p* < 0.001	*p* = 0.03	*p* < 0.001	*p* < 0.001
**Grade Concordance** **(FS/His)**				
Yes	74 (31%)	4 (22%)	13 (17%)	57 (40%)
No	11 (5%)	0	4 (5%)	7 (5%)
NA	154 (64%)	14 (78%)	61 (78%)	79 (55%)
*k* Cohen	*k* = 0.7	*k* = 1	*k* = 0.5	*k* = 0.8
*p* value	*p* < 0.001	*p* = 0.04	*p* = 0.01	*p* < 0.001

LS: Lynch Syndrome; LLC: Lynch-Like Cancer; MMR-proficient: mismatch repair-proficient; Bx: pre-operative biopsy; His: final histology; FS: frozen section; NA: not available; / not calculated.

**Table 5:** Clinical, surgical and pathological differences between MMR-deficient and MMR-proficient patients.

Characteristics	Total	MMR-Deficient	MMR-Proficient	*p* Value
	*n* = 239	*n* = 96	*n* = 143	
**BSO**				0.05
Yes	224 (94%)	94 (98%)	130 (91%)	
No	15 (6%)	2 (2%)	13 (9%)	
**Lymph Nodes Analysis**				0.01
Positive	160 (67%)	74 (77%)	86 (60%)	
Negative	79 (33%)	22 (23%)	57 (40%)	
LVSI				0.01
Yes	71 (30%)	37 (39%)	34 (24%)	
No	168 (70%)	59 (61%)	109 (76%)	
**Adjuvant Therapies**				0.005
Yes	113 (47%)	56 (58%)	57 (40%)	
No	126 (53%)	40 (42%)	86 (60%)	
**COC**				0.038
Yes	36 (15%)	20 (21%)	16 (11%)	
No	199 (83%)	76 (79%)	123 (86%)	
NA	4 (2%)	0 (0%)	4 (3%)	

MMR: mismatch repair; BSO: bilateral salpingo-ovariectomy; LVSI: lympho-vascular space invasion; COC: combined oral contraceptive; NA: not available.
